# Whole-Genome Sequence of “*Candidatus* Liberibacter solanacearum” Strain R1 from California

**DOI:** 10.1128/genomeA.01353-14

**Published:** 2014-12-24

**Authors:** Z. Zheng, N. Clark, M. Keremane, R. Lee, C. Wallis, X. Deng, J. Chen

**Affiliations:** a Department of Plant Pathology, South China Agricultural University, Guangzhou, Guangdong, China; b Department of Plant Sciences, California State University, Fresno, California, USA; c USDA-ARS, National Clonal Germplasm Repository for Citrus and Dates, Riverside, California, USA; d San Joaquín Valley Agricultural Sciences Center, Parlier, California, USA

## Abstract

The draft whole-genome sequence of “*Candidatus* Liberibacter solanacearum” strain R1, isolated from and maintained in tomato plants in California, is reported. The R1 strain has the genome size of 1,204,257 bp, G+C content of 35.3%, 1,101 predicted open reading frames, and 57 RNA genes.

## GENOME ANNOUNCEMENT

“*Candidatus* Liberibacter solanacearum” is an alphaproteobacterium associated with potato zebra chip (ZC), a recently emerging disease causing economic losses to the potato industry in North America (1). The bacterium also infects other solanaceous plants such as tomato (*Solanum lycopersicum*). The tomato/potato psyllid *Bactericera cockerelli* (Šulc) is responsible for transmission of the bacterium in the field. For this reason, the bacterial name “*Candidatus* Liberibacter psyllaurous” also was a proposed species name (2). First reported in Mexico in the 1990s, ZC is now widespread in the western and central United States, Mexico, Central America, and New Zealand (3). In California, ZC was first reported in the Lancaster area (4). “*Candidatus* Liberibacter solanacearum” currently cannot be cultured on artificial media. Characterization of this bacterium has relied heavily on genome analyses. The genome sequence of a Texas strain of “*Ca*. Liberibacter solanacearum” isolated from potato psyllid has been reported (5). Here, we report a draft whole-genome sequence of “*Ca*. Liberibacter solanacearum” strain R1 from a tomato plant in California.

“*Ca*. Liberibacter solanacearum” strain R1 was first identified in a tomato plant in Riverside, CA, and maintained in tomato plants through grafting in a greenhouse. DNA was extracted from infected tomato tissue using a GeneJET plant genomic DNA purification minikit (Thermo Fisher Scientific, Inc., Waltham, MA). The procedures of Zheng et al. (6) were then used to sequence “*Ca*. Liberibacter solanacearum” strain R1. In brief, bacterial DNA was enriched using a NEBNext microbiome DNA enrichment kit (New England BioLabs, Inc., Ipswich, MA), and total DNA was amplified using a REPLI-g minikit (Qiagen, Inc., Valencia, CA). Genome sequencing was carried out through the 454 GS-FLX system using Titanium chemistry (Roche, Branford, CT) and an Illumina MiSeq system (Illumina, Inc., San Diego, CA). The 454 sequencing yielded 100,867 reads with an average of 511 bp. The Illumina sequencing yielded 3.9 × 10^7^ reads with an average of 251 bp. “*Ca*. Liberibacter solanacearum” reads were filtered using stand-alone BLASTn (7) against the genome sequences of “*Ca*. Liberibacter solanacearum” (GenBank accession number CP002371), “*Ca*. Liberibacter asiaticus” (GenBank accession numbers CP001677, CP004005, and JFGQ00000000.1), “*Ca*. Liberibacter americanus” (GenBank accession number CP006604), and *Liberibacter crescens* (GenBank accession number CP003789). A total of 1,278,660 reads with an average of 250 bp were extracted using a Perl script. Assembly was performed using a combination of both Velvet 1/2/10 (https://www.ebi.ac.uk/~zerbino/velvet/) (8) and CLC Genomics Workbench 7.0. The draft whole genome of “*Ca*. L. solanacearum” strain R1 had 99 contigs (~250× coverage) ranging from 520 bp to 71,292 bp, adding to a total of 1,204,257 bp, with a G+C content of 35.3%. The R1 genome is 97% of the “*Ca*. Liberibacter solanacearum” ZC1 genome (1,258,278 bp). Annotation was conducted by using the RAST server (http://rast.nmpdr.org/) (9). The draft R1 genome was predicted to contain 1,101 open reading frames and 57 RNA genes.

### Nucleotide sequence accession numbers.

This whole-genome shotgun project has been deposited at DDBJ/EMBL/GenBank under the accession number JNVH00000000. The version described in this paper is version JNVH01000000.
